# The Association Between Work-Related Rumination and Executive Function Using the Behavior Rating Inventory of Executive Function

**DOI:** 10.3389/fpsyg.2020.00821

**Published:** 2020-05-19

**Authors:** Mark Cropley, Hannah Collis

**Affiliations:** ^1^School of Psychology, University of Surrey, Guildford, United Kingdom; ^2^Surrey Business School, University of Surrey, Guildford, United Kingdom

**Keywords:** work-related rumination, executive function, adults, workers, the BRIEF

## Abstract

Work-related rumination has been associated with a number of health complaints, however, little is known about the underlying factors associated with rumination. Previous work using proxy measures of executive function showed work-related rumination to be negatively associated with executive function. In this paper, we report two studies that examined the association between work-related rumination and executive function utilizing an ecological valid measure of executive function: the Behavior Rating Inventory of Executive Function (BRIEF-A, Roth et al., 2005). In study 1 (*N* = 63), high, relative to low work-related ruminators, were found to demonstrate lower executive function skills, in eight of the nine subscales of the BRIEF. The aim of study 2 (*N* = 237) was to identify, the key executive function subscale/s associated with work-related rumination. Controlling for known factors associated with work-related rumination (fatigue and sleep), regression analysis identified the behavioral regulation subscale “shift” as the key predictor within the model. Shift relates to our ability to switch attention, to think about different solutions, and dealing with and accepting change. It was concluded that these findings lend support for future research to develop interventions for enhancing shift ability, as an aid to reduce work-related ruminative thinking.

## Introduction

It is widely accepted that psychologically detaching from work—that is, switching off mentally from work—is crucial for fostering health and wellbeing (Wendsche and Lohmann-Haislah, 2017). Psychologically detaching from work has also been associated with greater productivity, engagement and creativity when employees return to work (Binnewies et al., 2010; Sonnentag and Kühnel, 2016; Vahle-Hinz et al., 2017). Switching off psychologically from work demands can been understood in terms of a continuum. At one end, a worker is completely mentally disengaged and detached from work, whilst at the other they constantly think and ruminate about work issues. Work-related rumination has been defined as a thought or thoughts directed to issues relating to work, that is/are repetitive in nature, and difficult to control (Cropley and Zijlstra, 2011).

There are many reasons why an individual may ruminate about work issues during their free time. For example, a worker may ruminate about having too much work to do, meeting an important deadline, or an unfinished task at work (Syrek et al., 2017). Workers may also ruminate about social issues at work, such as stress over a future meeting, or the perseveration of a negative comment by a colleague at work (Cropley and Millward, 2009).

Research has shown that ruminating outside of work is associated with a number of negative physical and psychological health outcomes, including increased risk of cardiovascular disease (Suadicani et al., 1993; Cropley et al., 2017), risk of stroke (Suadicani et al., 2011), increased cortisol secretion (Rydstedt et al., 2009; Cropley et al., 2015), negative mood (Pravettoni et al., 2007), exhaustion, sleep problems and fatigue (Cropley et al., 2006; Nylén et al., 2007). Furthermore, longitudinal data has also highlighted that work-related rumination dramatically increased exhaustion and reduced psychological well-being (Firoozabadi et al., 2018; Kinnunen et al., 2019). Additionally, it has been found that even with the use of emotional regulation strategies to deal with emotional exhaustion, work-related rumination mediates the relationship (Geisler et al., 2019).

Despite the wealth of literature surrounding the consequences of ruminative behavior, little is known about the mechanisms or factors associated in the process of ruminating. Here, we refer to mechanism in a general sense, as the system or factors working together, supporting the process through which rumination takes place, and aiming to answer the question of why people ruminate. Exploring this mechanism is important, as understanding the factors that are associated with, or predict rumination, will inform the design of future interventions aimed at helping individuals to stop ruminating about work. Recent research has started to examine potential cognitive processes associated with general rumination within the literature, and one which has attracted considerable attention is executive function.

Executive function is a theoretical construct relating to a set of cognitive processes that relate to how people manage and regulate their thoughts and behaviors. This construct has been defined by Diamond (2013) “*a family of top-down mental procedures that are necessary, when people have to pay or shift their attention in cases where intuition or automatic responses would be insufficient*.” Thus, executive function refers to the mental processes which are needed to concentrate and focus on activities (Diamond, 2013). Although there is debate concerning different types of executive function, it is acknowledged that there are three main functions: inhibition, working memory and cognitive flexibility (Miyake et al., 2000), which lead on to further higher order functions such as reasoning and planning (Collins and Koechlin, 2012; Diamond, 2013). Executive functions were presumed to primarily reside within the prefrontal cortex (Barrasso-Catanzaro and Eslinger, 2016), but it is now thought a variety of brain regions appear to underlie executive function (Munro et al., 2017). Regardless of location, deficiencies have been shown to result in various disorders and everyday problems (Hagen et al., 2016; Lantrip et al., 2016; Pope et al., 2017).

There are two contrasting theoretical approaches which support an interaction between rumination and executive functions. The Impaired Disengagement Hypothesis (Koster et al., 2011), argues that deficits in executive function (i.e., low levels of attentional control) increases the likelihood to ruminate (De Raedt and Koster, 2010; Koster et al., 2011). Relating this to the occupational setting, people who display poorer executive control could be more prone to making errors and mistakes at work, and therefore more likely to ruminate about them when not at work. Similarly, if people have depleted executive control, their mind is more likely to wander, and they will have more difficulty concentrating and focusing on tasks. Thus, a vicious cycle develops, where ruminative thinking is maintained by an impaired ability to exert control. The opposing view, the Resource Allocation Hypothesis, suggests that the association between rumination and executive function is due to rumination reducing executive function capacity (Levens et al., 2009). Once rumination is triggered, ruminative thought weakens cognitive performance by capturing attention and cognitive resources, thereby preventing these resources from being allocated to effortful tasks (Watkins and Brown, 2002). Relating this to the workplace, ruminating about work affects executive function, therefore reducing cognitive capacity, and places individuals at an increased risk of engaging in further ruminative thinking. And by continually ruminating, individuals have difficulty diverting their attention away from negative thoughts. Ruminating about work depletes executive resources leading workers to be less focussed and flexible in their thinking and cognition. This is in line with research showing that workers who ruminate are also at an increased risk of having accidents or making mistakes at work (Cropley et al., 2016).

Executive function is an abstract construct and is therefore fairly difficult to accurately assess, resulting in disagreement within the literature regarding the most effective approach. However, one of the most widely used interview measures for assessing executive function is the Behavior Rating Inventory of Executive Function (BRIEF-A, Roth et al., 2005). The BRIEF-A is an interview based self-report instrument developed to assess real-world manifestations of executive function in adults. The measure assesses nine subscales of executive function: Inhibit, Shift, Emotional Control, Self-monitor, Initiate, Working Memory, Plan/Organize, Task monitor, and Organization of materials, and from which are calculate three higher lever sub-indices of Behavioral Regulation (relating to how an individual’s controls their emotions, thoughts and behaviors), Metacognition (relating to planning, organization and working memory), and a combined Global Executive Composite (GEC) score.

In today’s competitive world, having high executive function skills are essential in the workplace. Deficits in any area of executive function—inhibition, cognitive flexibility, or working memory—can make it particularly difficult for workers to perform and complete tasks that require high level mental control. Indeed, a systematic review demonstrated a strong association between cognitive functions and job burnout (Deligkaris et al., 2014). To our knowledge however, only one paper has directly examined executive function and work-related rumination. In a series of three independent studies Cropley et al. (2016) reported that employees who ruminate about work report more cognitive failures, are less cognitively flexible and report less situational awareness at work. Despite the use of different methodologies—survey and interviews—and the generally supportive findings, the results are nonetheless limited as the authors used proxy measures of executive function.

The present paper reports two studies which aims to extend and advance the previous work by Cropley et al. (2016) to investigate the association between work-related rumination and executive functions.

## Study 1: Work Related Rumination and Executive Functions in Sales Professionals

The first study aimed to replicate the findings of Cropley et al. (2016), using the BREIF-A. Based on the aforementioned discussion, two hypotheses are proposed:

**H1:** High work-related ruminators would demonstrate lower Behavioral Regulation, Metacognition, and Global Executive Composite (GEC) scores, relative to low ruminators.
**H2:** High work-related ruminators would demonstrate lower executive function score in the nine subscales, relative to low ruminators.

### Method

Ethical approval from the University of Surrey ethical committee (NO. FT-1819-21) was obtained prior to data collection. One-hundred and four sales and recruitment professionals (52.9% males) completed this study, recruited via snowballing and opportunistic sampling methods. The mean age for this sample was 33.2 years (range 19–66 years, SD = 10.86), they had worked for their current company for between 1 month to 23 years (*M* = 5.87, SD = 6.94) and had been in the occupation of sales or recruitment for between 6 months to 31 years (*M* = 8.77, SD = 8.42). The majority of the sample occupied experienced, non-management positions (61%), with 11% in a management role, 12% in senior management, 8% entry level, and 7% in an administrative position. To answer the hypotheses, participants were categorized based on their responses to the affective rumination measure (see below) into two comparable groups. Those who scored 12 or less were categorized as low ruminators, whilst those who scored 16 or more were categorized as high ruminators (Querstret et al., 2016). These scores represented one standard deviation above and below the mean. The low ruminator group consisted of 17 males and 11 females, with ages ranging from 20 to 55 (*M* = 34.71, SD = 12.03). The high ruminator group consisted of 16 males and 19 females, with ages ranging from 22 to 66 (*M* = 30.74, SD = 10.15). These participants were then selected to be interviewed using the BRIEF-A and are subsequently included in the analysis.

### Measures

#### Work-Related Rumination

The affective rumination subscale of the Work-Related Rumination Questionnaire (WRRQ; Cropley et al., 2012) was used to determine individuals’ levels of affective work-related rumination. The 5 items are scored on a 5-point Likert scale in response to statements, for example “Do you become tense when you think about work related issues in your free time?” with the option to select “Very Seldom/Never,” “Seldom,” “Sometimes,” “Often” and “Very Often/Always” for each statement. The WRRQ has been shown to have good reliability and validity and has been successfully used within a number of previous studies (for example Syrek et al., 2017; Querstret et al., 2016) and has a Cronbach’s alpha reliability of 0.87 within this sample.

#### Executive Function

The BRIEF-A (Roth et al., 2005) consists of 75 questions and produces an overall score of executive function (Global Executive Composite, GEC), which is comprised of two index scores: Behavioral Regulation Index (BRI) and the Metacognition Index (MI). The BRI (α = 0.91) is formed of four subscales: Inhibit (8 items, e.g., “I tap my fingers of bounce my legs,” α = 0.75), Shift (6 items, e.g., “I have trouble thinking of a different way to solve a problem when stuck,” α = 0.73), Emotional Control (10 items, e.g., “I have angry outbursts,” α = 0.90) and Self-monitor (6 items, e.g., “I talk at the wrong time,” α = 0.73); while the MI (α = 0.93) is formed of five scales: Initiate (8 items, e.g., “I have trouble getting ready for the day,” α = 0.78), Working Memory (8 items, e.g., “I forget what I am doing in the middle of things,” α = 0.83), Plan/Organize (10 items, e.g., “I get overwhelmed by large tasks,” α = 0.80), Task Monitor (6 items, e.g., “I make careless errors when completing tasks,” α = 0.73), and Organization of Materials (8 items, e.g., “I am disorganized,” α = 0.82). Participants are presented with a list of statements and asked if they have been a problem “Often,” “Sometimes” or “Never” over the past month, relating to all aspects of life, including home, work and leisure. The raw scores are transformed into T scores in comparison to normative samples (Roth et al., 2005), with a score of 50 representing the normative mean. Therefore, higher scores indicate poorer executive functions. The BRIEF-A is used as a diagnostic tool for cognitive disorders related to executive functions, it is considered to be an ecologically valid measure of executive function, and it has been utilized in a number of studies (for example Hagen et al., 2016; Pope et al., 2017). The overall GEC Cronbach’s alpha of this measure is 0.88 within this sample.

### Results

A Multivariate Analysis of Variance (MANOVA) was conducted to detect any initial effects of rumination on the three main dependent variables between groups: Global Executive Composite (GEC), Behavioral Regulation (BRI) and Metacognition (MI). Using Pillai’s trace, there was a significant effect of rumination level on each broad construct within the BRIEF, *F*(6,132) = 3.33, *p* = 0.004, *V* = 0.263, partial η^2^ = 0.13. Separate ANOVAs were then conducted to examine significant differences between the high and low ruminators on each sub-measure of executive functions. Due to the number of tests performed, significance was accepted at 0.01 or higher. Age and gender were tested as covariates, however, there was no effect found and so were excluded from further analysis.

Table 1 displays the results of the ANOVAs, means, standard deviations and effect-sizes (η^2^) for the GEC, BRI and MI T scores. As demonstrated, all three factors were statistically significant, with poorer executive function (higher scores) reported in the high rumination group. Therefore, the first hypothesis is supported. To analyze the individual executive functions, further analysis revealed significant differences for each of the nine subscales T scores, except for the self-monitoring item (see Table 2). Overall, these findings demonstrate that higher levels of work-related rumination are associated with poorer executive functions globally, impacting upon both behavioral facets of executive functions and the cognition facets, and further supporting the proposed hypotheses.

**TABLE 1:** T-Score means, standard deviations, and ANOVA results for Behavioral Regulation Index, the Metacognition Index, and the combined Global Executive Composite (GEC) by rumination group.

	Low ruminators		High ruminators				
	Mean	SD	Mean	SD	*F*	η^2^	*p*
Behavioral Regulation Index	47.75	1.61	56.40	1.44	15.88	0.20	0.001
Metacognition Index	46.78	1.65	55.82	1.48	16.53	0.21	0.001
Global Executive Composite	47.07	1.54	56.17	1.38	19.18	0.23	0.001

*N = 63.*

**TABLE 2:** T-Score means, standard deviations and ANOVA results for the subscale measures of the BRIEF-A, separated by rumination group.

	Low ruminators		High ruminators				
	Mean	SD	Mean	SD	*F*	η^2^	*p*
Inhibit	50.71	1.80	59.91	1.61	14.40	0.19	0.001
Shift	49.64	1.82	56.45	1.63	7.75	0.11	0.007
Emotional control	46.71	1.84	54.34	1.64	9.55	0.13	0.003
Self-monitor	47.71	1.70	51.51	1.52	2.77	0.04	ns
Initiate	45.57	1.71	52.91	1.52	10.25	0.14	0.002
Working memory	49.35	2.00	58.97	1.79	12.78	0.17	0.001
Plan/organize	49.71	1.63	55.62	1.46	7.23	0.10	0.009
Task monitor	47.53	1.70	55.77	1.52	13.02	0.17	0.001
Organization of materials	44.21	1.65	50.17	1.48	7.17	0.10	0.001

*N = 63.*

## Study 2: Affective Rumination, Executive Functions and Job Demands

Having supported our first two hypotheses, the second question to address is: what are the key executive functions associated with work-related rumination? For this study we treated work-related rumination as the dependent variable and examined the subscales of the BRIEF to identify the most predictive subscale. The rationale for this switch in methodological design, is that this research is aiming to first identify an association, rather than establishing a cause-consequence direction. In analyzing the studies from both perspectives allows this contribution to remain open in the debate concerning directionality (see Discussion). As executive function and rumination have both been associated with fatigue and sleep (Joyce et al., 1996; Van der Linden et al., 2003; Durmer and Dinges, 2005; Nilsson et al., 2005; Thomas, 2005; Nylén et al., 2007; Berset et al., 2011; Plessow et al., 2011; Querstret and Cropley, 2012; Diamond, 2013), we controlled for the effects of fatigue and sleep in the analysis. Similarly, it has been established that there is an association between work-related rumination and job demands (Cropley and Millward-Purvis, 2003; Perko et al., 2017; Querstret and Cropley, 2012) and gender (Rydstedt et al., 2009), so these variables were controlled within the regression model. No specific hypothesis was made.

### Method

This study was pre-registered on Aspredicted.org (#16857). The same sampling methods produced a novel sample of 237 (61.6% female) working individuals. Their ages ranged between 19 and 66 (*M* = 33.8, SD = 12.7). The sample was predominantly White British in ethnicity (83.5%). All participants were in full time employment, with 17.3% at entry level, 18.1% intermediate non-management, 24.1% experienced non-management, 17.3% first level management, 12.2% middle level management and 11% upper management. This sample hailed from a number of occupations, including 17% from healthcare, 11% accountancy and finance, 9% recruitment or human resources, 7% education and 6% from business.

### Measures

Work-related rumination and executive function were assessed using the measures reported in Study 1. The reliability alphas for all time two variables are presented in Table 3.

**TABLE 3:** Correlations for study two variables.

	*M*	SD	1	2	3	4	5	6	7	8	9	10	11	12	13	14	15	16	17	18	19
1. Age	33.80	12.72	–	–	–	–	–	–	–	–	–	–	–	–	–	–	–	–	–	–	–
2. Gender	1.62	0.49	0.10	–	–	–	–	–	–	–	–	–	–	–	–	–	–	–	–	–	–
3. Job level	3.54	1.82	0.49**	0.03	–	–	–	–	–	–	–	–	–	–	–	–	–	–	–	–	–
4. Fatigue	18.71	6.91	0.21**	0.06	0.16*	(0.94)	–	–	–	–	–	–	–	–	–	–	–	–	–	–	–
5. Job demands	34.45	4.05	0.30**	0.18**	0.23**	0.16*	(0.73)	–	–	–	–	–	–	–	–	–	–	–	–	–	–
6. Sleep	5.55	3.01	0.09	−0.02	0.18**	0.42**	−0.02	(0.75)	–	–	–	–	–	–	–	–	–	–	–	–	–
7. WRAR	14.42	4.49	0.17**	0.19**	0.17**	0.60**	0.24**	0.43**	(0.90)	–	–	–	–	–	–	–	–	–	–	–	–
8. GEC	51.90	9.31	0.13	−0.02	0.13*	0.48**	0.06	0.52**	0.44**	(0.96)	–	–	–	–	–	–	–	–	–	–	–
9. BRI	52.00	10.96	0.05	0.04	0.12	0.44**	0.04	0.48**	0.44**	0.90**	(0.92)	–	–	–	–	–	–	–	–	–	–
10. MI	51.11	9.04	0.31**	−0.02	0.20**	0.48**	0.14*	0.49**	0.41**	0.92**	0.69**	(0.94)	–	–	–	–	–	–	–	–	–
11. Inhibit	52.72	10.26	−0.03	−0.15*	0.04	0.30**	0.06	0.29**	0.22**	0.75**	0.77**	0.60**	(0.73)	–	–	–	–	–	–	–	–
12. Shift	52.30	9.87	0.07	0.04	0.11	0.42**	0.00	0.43**	0.49**	0.72**	0.75**	0.59**	0.43**	(0.74)	–	–	–	–	–	–	–
13. Emotional control	51.61	11.53	0.05	0.19**	0.09	0.40**	0.03	0.46**	0.43**	0.74**	0.88**	0.52**	0.48**	0.63**	(0.93)	–	–	–	–	–	–
14. Self-monitor	47.62	9.60	−0.01	−0.13	0.06	0.20**	0.02	0.23**	0.17**	0.69**	0.72**	0.53**	0.64**	0.38**	0.48**	(0.78)	–	–	–	–	–
15. Initiate	51.91	10.94	0.18**	−0.05	0.18**	0.48**	0.07	0.53**	0.38**	0.83**	0.66**	0.86**	0.53**	0.59**	0.55**	0.45**	(0.78)	–	–	–	–
16. Working memory	55.11	11.21	0.16*	0.09	0.14*	0.40**	0.07	0.39**	0.37**	0.79**	0.64**	0.80**	0.55**	0.60**	0.49**	0.44**	0.63**	(0.78)	–	–	–
17. Plan/organize	51.94	10.11	0.18**	−0.09	0.12	0.43**	0.09	0.45**	0.33**	0.86**	0.65**	0.91**	0.58**	0.57**	0.47**	0.53**	0.79**	0.67**	(0.83)	–	–
18. Task monitor	52.65	10.83	0.17**	−0.10	0.07	0.41**	0.14*	0.35**	0.33**	0.78**	0.58**	0.84**	0.54**	0.46**	0.40**	0.50**	0.64**	0.67**	0.75**	(0.72)	–
19. Organization	48.67	10.41	0.18**	−0.02	0.10	0.26**	0.10	0.35**	0.25**	0.68**	0.45**	0.77**	0.44**	0.31**	0.34**	0.40**	0.58**	0.46**	0.66**	0.57**	(0.84)

N = 237. Gender: Males = 1, Females = 2. T-scores are reported for the BREIF-A variables. WRR = Work-Related Rumination. Reliability alphas presented in parenthesis on the diagonal. *p < 0.05 **p < 0.01.

#### Job Demands

Eleven items previously selected by Querstret and Cropley (2012) were taken from the Job Content Questionnaire (JCQ; Karasek et al., 1998). Items, such as “Do you have to work very fast?” and “Is your job boring?” (reversed item) are scored on a 4-point Likert scale, ranging from “1 Never/almost never” to “4 Often.” Higher scores are indicative of increased job demands.

#### Fatigue

The present study employed the 15 item Occupational Fatigue Exhaustion Recovery scale (OFER; Winwood et al., 2006) as a workplace focused measure of fatigue. Items, such as “I often feel I’m “at the end of my rope” with my work” and “My work drains my energy completely every day” are responded to on a 7-point Likert scale, ranging from “strongly disagree” to “strongly agree.”

#### Sleep

Sleep was assessed using the Pittsburgh Sleep Quality Index (PSQI; Buysse et al., 1989). This 19 item scale results in a global sleep score, comprised of seven factors (daytime dysfunction, sleep duration, sleep latency, habitual sleep efficiency, sleep disturbances, use of medication and subjective sleep quality), which ranges from 0 to 21, with scores above 5 indicating poor sleep.

### Results

Descriptive and bivariate correlations between the variables are presented in Table 3. As can be seen within the table, both fatigue and sleep are strongly positively correlated with rumination, *r* = 0.60, *p* < 0.001 and *r* = 0.43, *p* < 0.001 respectively, as well as the executive function subscales. Interestingly while job demand is correlated with rumination, *r* = 0.24, *p* < 0.001, it is only correlated with one subscale of executive function: task monitoring, *r* = 0.14, *p* = 0.04. Regarding the correlations between the executive function scales, the highest correlations were between the subscales and the related index, which would be expected based on the scoring system. All other correlations are below 0.7, suggesting collinearity between variables unlikely (Berry and Feldman, 1985).

Multiple regression analysis was conducted to identify the key predictor of work-related rumination from the nine subscales of the BRIEF-A. In addition to gender and job demands, we also controlled for age and job level in the analysis due to correlating with both executive function and WRR. The individual control variables were entered in step 1, job demands, fatigue, and sleep were entered in step 2, and the predictor executive function variables were entered in step 3. The results of the analysis are displayed in Table 4. The final model is significant, *F*(15,220) = 15.10, *p* < 0.001, *R*^2^ = 0.507, showing that executive functions predict levels of work-related rumination, accounting for over 50% of the variance. Within this final model, fatigue (*t* = 7.08, *p* < 0.001, β = 0.41), sleep (*t* = 2.81, *p* = 0.005, β = 0.17), job demands (*t* = 3.22, *p* = 0.001, β = 0.17) and the executive function subscale shift (*t* = 4.20, *p* < 0.001, β = 0.30) were the significant predictor variables. Thus, the inability to shift or change one’s thinking was the key executive function associated with work-related rumination.

**TABLE 4:** Multiple regression results for predicting work-related affective rumination.

	Step 1		Step 2		Step 3	
	β (SE)	*t*	β (SE)	*t*	β (SE)	*t*
Gender	0.175 (0.586)	2.75**	0.137 (0.461)	2.73**	0.102 (0.492)	1.90
Age	0.096 (0.026)	1.31	−0.017 (0.021)	−0.29	−0.011 (0.021)	−0.19
Job level	0.118 (0.179)	1.63	0.025 (0.142)	0.44	0.023 (0.139)	0.41
Fatigue	–	–	0.471 (0.036)	8.47***	0.412 (0.038)	7.08***
Sleep	–	–	0.231 (0.074)	4.19***	0.169 (0.081)	2.81**
Job demands	–	–	0.154 (0.059)	2.90**	0.167 (0.057)	3.22**
Inhibit	–	–	–	–	−0.050 (0.031)	−0.71
Shift	–	–	–	–	0.300 (0.032)	4.20***
Emotional control	–	–	–	–	0.050 (0.028)	0.69
Self-monitor	–	–	–	–	−0.013 (0.031)	−0.19
Initiate	–	–	–	–	−0.68 (0.036)	−0.78
Working memory	–	–	–	–	−0.012 (0.029)	−0.17
Plan/organize	–	–	–	–	−0.142 (0.044)	−1.43
Task monitor	–	–	–	–	0.082 (0.034)	1.01
Organization	–	–	–	–	0.081 (0.028)	1.23
Constant	(1.212)	7.96***	(−1.111)	−0.56	(−5.119)	−2.16*
*F*	–	5.73***	–	30.71***	–	15.10***
*R*^2^	–	0.069	–	0.446	–	0.507
Adjusted *R*^2^	–	0.057	–	0.431	–	0.474
Δ*F*	–	–	–	51.93***	–	3.04***
Δ*R*^2^	–	–	–	0.377	–	0.061

N = 237. Values in parentheses represent standard error. ΔF and ΔR^2^ report changes from Step 1. *p < 0.05; **p < 0.01; ***p < 0.001.

## Discussion

It is estimated that around a third of the population have difficulty mentally disengaging from work (Gallie et al., 1998; Cropley and Zijlstra, 2011). And, as work-related rumination has been associated with a range of health problems, studies are needed to understand the cognitive mechanisms that influence the recovery process. To our knowledge, this is the first paper to examine the association between executive function and work-related rumination using a fully validated measure and within two separate samples.

The results of study 1, were consistent with previous research (Cropley et al., 2016), and demonstrated that high work-related ruminators had poorer executive function skills, relative to low ruminators. This finding was consistent across the three global executive function groups (Behavioral Regulation Index, Metacognition Index, and the Global Executive Composite group scores), and eight of the nine subscales. The only subscale not associated with work-related rumination was self-monitoring, and we speculate that perhaps this may have been due to the sample population. The sales industry is a fairly unique environment due to the fast paced, high pressure and opportunistic nature of the work. Sales staff in the present study are consistently encouraged to perform to goals and are trained to monitor their behavior, so it seems perfectly reasonable for them to be particularly good at self-monitoring. It was therefore deemed important to recruit workers from different professions for study 2.

Study 2 further supported the association between executive function and work-related rumination. Interestingly, within the regression model, Shift appeared to be the most important predictor. The subscale Shift relates to our ability to switch attention, to think about different solutions or ways of thinking, and dealing with and accepting change. This is an interesting finding as within the literature it is the function of inhibition which is the most cited executive functions in the relationship with rumination. Indeed, a negative association between rumination and inhibition has been shown in several clinical and experimental studies (Berman et al., 2011; Fawcett et al., 2015; Mor and Daches, 2015), although the research here is somewhat inconsistent, as at least four types of inhibition have been associated with rumination (viz. inhibition of distracting information, inhibition of no longer relevant information, proponent response inhibition and task switching inhibition; De Lissnyder et al., 2011; Colzato et al., 2018; Owens and Derakshan, 2013; Whitmer and Banich, 2007; Zetsche et al., 2012). Despite this disparity between our findings and those within the clinical literature, our findings are broadly in line with those of Yang et al. (2017), whose meta-analytic review reported significant associations between rumination and the functions of inhibition and shift. Interesting to note, their review found no significant differences for working memory in relation to rumination, whereas study 1 in the present study did, with high ruminators reporting poorer memory, relative to the controls. The general differences between the findings of study 1 and 2, and previous research may be due to the focus on work-related rumination, as opposed to general or depressive rumination, within the present studies. Working memory includes working with and manipulating information in the mind (Diamond, 2013), which would be much more applicable to tasks performed in the work environment in comparison to general life and interpersonal interactions. While this could indicate that work-related rumination shares many qualities to more general ruminative thinking, these slight divergences suggest a different process, and would therefore require different solutions to treat work-related rumination.

There were a number of novel aspect and strengths of the present studies. It was the first, to our knowledge, to assess the association between executive function and work-related rumination using a validated measure of executive function. Secondly, we controlled for fatigue, sleep, and job demands, which are well known factors that can modify rumination and executive function. Thirdly, the study had ecological validity and utilized individuals from real-life settings with a reasonable sample size.

There were however, some limitations, and issues we could not address. The findings presented here are cross-sectional due to the nature of the research question under investigation. It was therefore not the focus of the present studies to investigate claims of causality between work-related rumination and executive functions. As reported in the introduction, within non-work-related samples however, the existent literature on this topic is greatly mixed, with some authors suggesting that rumination is a result of deficits in executive function (Linville, 1996; Koster et al., 2011), while others propose that rumination depletes resources and limits the ability to be cognitively flexible, severely impairing broader executive functions (Watkins and Brown, 2002; Philippot and Brutoux, 2008). It is however, entirely plausible that causality works both ways in a reciprocal relationship. More research needs to be conducted here to provide clarity to this question as a result of the present findings. Secondly, whilst the use of convenience sampling methods here did provide a fairly representative insight into a variety of professions and industries within the United Kingdom, we encourage caution when generalizing the results. Another issue centers on the instrument used to assess executive function. Whilst the BREIF-A is indeed a validated and effective measure of executive function it nonetheless relies on self-reporting. Future research could/should employ more objective, behavioral measures to substantiate the current results.

Notwithstanding, the findings of the present studies may be utilized to inform the development of interventions. Work-related rumination and deficits in executive functions are considered to be well-established risk factors leading to profound and debilitating mental and physical health problems, reduced work performance and quality of life. Given these costs, there is a pressing need to develop cost-effective, parsimonious interventions which have a strong theoretical and empirical basis. It has been noted that executive functions are trainable (Diamond, 2013). If one accepts the premise that rumination and executive functions are mechanisms of each other, then interventions targeted at one could potentially impact the other. However, prior literature has only explored interventions aimed solely at each variable. For example, exercise has been found to increase executive functioning (Guiney and Machado, 2013; Dupuy et al., 2015), as well as directly targeted function training, such as inhibition training, which has proven to be successful in directing attention (Daches and Mor, 2014). Rumination interventions, on the other hand, have been more focused on controlling/distracting thoughts and behaviors in general, either through CBT training, mindfulness (Hahn et al., 2011; Hülsheger et al., 2014; Querstret et al., 2016) or breathing and meditation (Plans et al., 2019). The lack of successful evidence-based interventions is perhaps a consequence of utilizing existing descriptive theories within the field, and the tendency to take a broad approach, which lacks insight and understanding into the actual underlying mechanisms of rumination. Perhaps, the ideal approach would be to target both the symptoms of rumination and the underlying mechanisms, using a two-pronged intervention approach.

## Conclusion

There is increasing awareness of the importance of unwinding and switching off from work, and that thinking and ruminating about work can impede the recovery process. In this paper, we presented two distinct studies that demonstrated work-related rumination to be associated with reduced executive function. We were not able to make any causal inferences and further work is needed to establish causality; nonetheless, these findings add to our understanding about the mechanisms underlying work-related rumination and may be used to inform future interventions.

## Data Availability Statement

The datasets generated for this study are available on request to the corresponding author.

## Ethics Statement

The studies involving human participants were reviewed and approved by the University of Surrey Ethics Committee. The patients/participants provided their written informed consent to participate in this study.

## Author Contributions

HC and MC designed both studies, and equally involved in the write-up of the article. HC responsible for data collection in study 1 and management of study 2 data collection.

## Conflict of Interest

The authors declare that the research was conducted in the absence of any commercial or financial relationships that could be construed as a potential conflict of interest.
